# Family functioning, marital quality and social support in Chinese patients with epilepsy

**DOI:** 10.1186/s12955-015-0208-6

**Published:** 2015-01-28

**Authors:** Yi-he Wang, Michelle Haslam, Ming Yu, Juan Ding, Qian Lu, Fang Pan

**Affiliations:** Department of Medical Psychology, Shandong University School of Medicine, 44#, Wenhua Xi Road, Jinan, Shandong 250012 China; Culture & Health Research Center, Department of Psychology, University of Houston, 126 Heyne Building, Houston, TX 77204 USA; Department of epilepsy, The Second Affiliated Hospital of Shandong Traditional Chinese Medicine College, Jinan, 250001 Shandong China

**Keywords:** Family function, Marital quality, Epilepsy, Social support, Depression, Anxiety

## Abstract

**Background:**

The purpose of this study was to examine family functioning, marital quality, social support, and anxiety and depression in Chinese patients with Epilepsy (PWE) in comparison with healthy people.

**Methods:**

This case–control study included 42 PWE and 42 healthy controls. Participants completed the Zung’s self-rating depression scale, the Zung’s self-rating anxiety scale, the Chinese version of family cohesion and flexibility evaluation scales, the Chinese version of the marital inventory ENRICH, and the Chinese versions of the social support rating scale and perceived social support scale.

**Results:**

PWE reported higher levels of anxiety and depression, and lower levels of family cohesion, marriage quality and social support compared with controls. Support within and outside the family was negatively associated with depression, however social support did not significantly predict depression in PWE. In patients, support within the family and emotional support predicted family cohesion and marriage quality. Instrumental support was negatively associated with anxiety in patients but positively associated with depression in healthy controls. Support within the family predicted family cohesion and marriage quality in both the control group and patient group, depression predicted family adaptation in both the control group and patient group, while support outside the family predicted marriage quality only in the patient group. Both emotional and instrumental support predicted family adaptation in the control group, and emotional support predicted family cohesion in patients.

**Conclusions:**

PWE in China had higher levels of anxiety and depression, dissatisfaction with family functioning and marital life, and less social support compared with healthy controls. Emotional support within and outside families promoted family cohesion and marriage quality, depression decreased family adaptation, and instrumental support decreased anxiety of PWE. These findings suggest that enhancing family and emotional supports and decreasing depression could promote the family functioning and marital quality of PWE, and instrumental support may play a role in decreasing anxiety.

## Background

Epilepsy is a chronic neurological disorder characterized by seizures originating from abnormal electrical signals in the brain [1]. About 50 million people worldwide have epilepsy, and nearly 80% of epilepsy occurs in developing countries [2]. Although clinical primary treatment goals are focused on the physical complications of epilepsy through seizure control [1,3,4], patients with epilepsy (PWE) face many other challenges, including psychological difficulties (e.g. lower self-esteem, depression and anxiety) and social complications (e.g. driving restrictions, unemployment and social isolation) [5-8]. Recent research has suggested that PWE have culturally sensitive problems due to the longevity of the disorder. Associated morbidity, treatment gap and negative public attitudes towards epilepsy exist in different countries [2,9], all of which can have a negative impact on quality of life.

Greater social support has been linked to better quality of life in patients with chronic diseases [10,11]. Social support is defined as the perception that an individual is a member of a network in which one can give and receive help, affection and obligation [12]. Social support can be received from family members, friends, colleagues, and medical personnel [12]. However, studies have revealed that the outcomes of social support are dependent on the source and the kind of support. Emotional support coming from family members has been found to have positive effects on mental health, family cohesion and marriage quality while support coming from people outside the family and instrumental support had a negative effect on family and marriage variables [13]. In a laboratory setting, it has been found that support from close friends decreases cardiovascular reactivity but support from acquaintances or strangers increases cardiovascular reactivity when individuals are facing a stressor [14]. These results indicate that social support has complicated and diverse effects. Research about epilepsy patients’ interactions with society has indicated that their interactions are often negatively affected by the consequences of epilepsy [12,15-17]. Social relation deficits have been found to be associated with poorer quality of life in PWE [16]. These results suggest that it is necessary to examine the effect of social support on variables of QOL including family and marital life.

Family functioning includes family cohesion and flexibility dimensions according to Olson’s circumflex model of family functioning [18]. Family cohesion refers to the emotional bonds between family members and family flexibility refers to the quality and expression of the family’s leadership, organization, roles, and relationship rules. Well-functioning families are considered balanced, falling mid-range on each dimension. Poorly functioning families are considered unbalanced on these dimensions, falling either low (e.g., disengaged, rigid) or high (enmeshed, chaotic) on these characteristics. Family functioning is expected to shift along the dimensions in predictable ways during the life cycle and in response to stress [19,20]. The conception of marriage quality is related to the couple’s subjective satisfaction of the relationship [21]. One study suggested that people with active epilepsy encountered more marital discord than controls and depression and social support satisfaction were significant predictors of marital adjustment [22]. Other studies have found that family support contributed significantly to QOL of PWE and patients’ QOL also depended significantly on the QOL of the family members [23,24], suggesting a strong association between social support and QOL in both PWE and their family members respectively. Family functioning may be an important treatment target to enhance coping in PWE [25], however few studies have explored the association between family functioning and social support of PWE in China.

The current study aimed to examine differences in depression and anxiety, family functioning, marital quality and social support between Chinese married PWE and healthy controls. Based on previous findings that epilepsy and epileptic seizures are negatively associated with quality of life [5-8], and positively associated with higher levels of anxiety and depression [5,22,25,26], it was hypothesized that patients would experience more anxiety and depression, and have lower family functioning and marriage quality compared with healthy controls (hypothesis 1). Previous research has found that those with chronic illness are often more dissatisfied with their relationships and feel more isolated from others [10]. Therefore it was hypothesized that PWE would report receiving less social support than healthy controls (hypothesis 2). Due to the fact that social support includes instrumental support, emotional support, and social support from both those within and outside of the family, this study explored whether the type and sources of social support, as well as anxiety and depression influenced family function and marital quality (hypothesis 3).

## Methods

### Participants and protocol

Approval for the study was obtained from the Institutional Ethics Committee of Medical College of Shandong University and informed consent was given by each subject. The study group consisted of 84 participants including 42 married PWE and 42 married healthy individuals from the urban area of Jinan in the Shandong Provence. Patients had been correctly diagnosed [27] and they were outpatients from the Department of Epilepsy, Second Affiliated Hospital of Shandong University of Traditional Chinese Medicine from March 2012 to July 2013.

Inclusion criteria included: (1) a diagnosis of epilepsy received at least 24 months prior; (2) married at least 1 year prior; (3) typical clinical seizures; and (4) seizures well controlled by drug treatments. Exclusion criteria included: (1) unmarried; (2) insufficient knowledge of written or spoken Chinese language; (3) symptomatic epilepsy; (4) the presence of a chronic psychiatric condition (except for depression or anxiety); and (5) the presence of other life-threatening co-morbidities (e.g., cancer or multiple organ system failure). Healthy people in the control group were recruited through advertisements in the health center of the same hospital. They were matched based on gender, age, educational level, and employment level of the patient groups. Patients were informed about the study during a medical appointment, and took the questionnaires home to complete and return. Healthy controls completed the questionnaires at the same time as a health survey was administered.

### Measurements

#### Depression and anxiety

Chinese versions of the Zung’s Self-Rating depression Scale (SDS-CV) [28] and The Zung’s Self-Rating Anxiety Scale (SAS-CV) [29] were used to measure depression and anxiety. SDS-CV and SAS -CV are both 20-item questionnaires, and items are answered on a 4-point Likert scale, ranging from 1 to 4. Higher scores indicate more depression and anxiety. The internal consistency (alpha) of SDS-CV is 0.92. The correlation coefficient between SAS-CV and Hamilton Anxiety Scale is 0.365.

### Family functioning and marital quality

The Chinese version of the Family Adaptability and Cohesion Scales (FACES II-CV) was used to rate actual experience of family function [30]. There are two subscales of FACES II-CV, one that assesses family adaptability and one that assesses cohesion. Family adaptability is the ability of the family system to adjust its rules, provide structure and adjust relationship patterns in response to changes; the subscale includes items such as: ‘Each family member is involved in making major family decisions’. Family cohesion is considered the emotional bonding that family members have with each other; this dimension includes items such as ‘family members try to support each other when the family faces difficulty’. The items are rated on a five-point likert scale, with moderate scores in cohesion and flexibility sub-dimensions indicating a balanced family and extremely low or high scores indicating an imbalanced family. Test-retest reliability ranged from 0.84 to 0.91 and Cronbach’s alpha coefficients were 0.85 and 0.73, respectively.

The Chinese version of the ENRICH marital inventory (Evaluating & Nurturing Relationship, Issues, Communication & Happiness) was used to judge the degree of Marital quality [21]. ENRICH includes 12 factors: marital satisfaction, personality compatibility, the couple exchange, conflict resolution, economic arrangements, leisure activities, sexual life, children and marriage, the relationship between friends and relatives and the role of equality and belief consistency. Global measurement of the 12 factors was used in the present study, with a higher score indicating better marital quality. The validity and reliability of ENRICH shows internal consistency (alpha) ranging from 0.68 to 0.86 in every dimension and test-retest correlation coefficients range from 0.77 to 0.96. The discriminant reliability ranges from 85% to 90%.

### Social support

The Chinese versions of the Social Support Rating Scale (SSRS) and Perceived Social Support Scale (PSSS) were used in the present study [31,32]. SSRS divides social support into instrumental support and emotional support. Instrumental support is defined as objective and visible help, including material assistance and financial support. Emotional support is the social state of the individual’s emotional experiences and satisfaction [31]. Higher scores indicate stronger social support. The SSRS has been used with in a wide range of Chinese populations due to its high reliability and validity [33-35], with two month test-retest reliability of 0.92. The PSSS emphasizes the individual’s perceived social support from various sources, such as family members, relatives and friends. There are two dimensions in PSSS: support within the family and support outside the family. Cronbach’s alpha coefficients is 0.91 and test-retest reliability is 0.85 [32].

### Statistical analysis

Statistical analyses were carried out using SPSS version 18.0 (SPSS Inc., Chicago, USA). Univariate relationships were determined between sociodemographics; mental scales scores in two groups were analyzed using independent sample t tests. Pearson’s correlations were used to determine the associations between depression, anxiety, social supports, family functioning and marital quality. Multi-linear regression analyses were used to determine the different kinds and sources of social support and negative emotion affecting family functioning and marital quality. The different kinds of social support, anxiety and depression were entered as independent variables and family cohesion, family adaptation and marital quality were entered as dependent variables. Separate regression analyses were conducted in patients and healthy control samples, one for each domain of variables. All statistical tests were two-tailed, and p <0.05 was regarded as being statistically significant.

## Results

### Comparison of demographic characteristics of two groups

No significant differences emerged for demographic characteristics between PWE and healthy controls (Table 1).

**Table 1:** **Demographic characteristic of the subjects (n = 84)**

**Items**	**Control group (n = 42)**	**PWE group (n = 42)**	***F*** **/χ** ^**2**^	***p***
Age (year) ± SD	42.31 ± 13.50	41.62 ± 13.38	0.041	0.840
Male/female	22/20	22/20	0.243	0.624
Education			0.091	0.602
Middle school graduate	18 (42.85%)	19 (45.23%)		
High school graduate	16 (38.09%)	16 (38.09%)		
College graduate	8 (19.04%)	7 (16.67%)		
Employment status			1.048	0.380
Employment	41 (97.62%)	39 (92.85%)		
Unemployment	1 (2.38%)	3 (7.14%)		

### Depression and anxiety, family functioning, marriage quality and social support

PWE had higher levels of anxiety and depression compared with healthy controls. PWE reported less family cohesion and marriage quality than healthy controls. No differences in family adaptation were found between the two groups. Furthermore, patients reported that they received less emotional support and instrumental support compared with the control group (Table 2).

**Table 2:** **Negative emotion, family functioning, marriage quality and social support in two groups**

**Measure**	**Subscale**	**Control group (n = 42)**	**Patients group (n = 42)**	***t***	***p***
**SAS-CV**	Anxiety	28.28 ± 5.64	42.66 ± 8.86	−2.356	0.022
**SDS-CV**	Depression	40.81 ± 7.76	47.72 ± 10.04	−3.078	0.003
**FACES II-CV**	Family cohesion	73.78 ± 8.62	56.75 ± 9.44	7.534	<0.001
	Family adaptation	48.00 ± 8.87	45.75 ± 10.30	0.936	0.535
**ENRICH**	Marriage quality	347.03 ± 34.27	324.75 ± 39.63	2.045	0.019
**PSSS**	Support within family	16.22 ± 4.20	15.19 ± 4.62	0.934	0.354
	Support outside family	28.69 ± 7.45	28.53 ± 7.98	0.081	0.934
**SSRS**	Instrumental support	10.47 ± 2.82	7.38 ± 1.94	5.097	<0.001
	Emotional support	23.69 ± 5.91	18.72 ± 4.40	3.814	<0.001

### Correlations between family functioning, marriage quality, negative emotion and social support

Table 3 shows the results of the correlations between the SDS-CV, SAS-CV, FACES II-CV, ENRICH, SSRS and PSSS scales. Support within the family was positively associated with family cohesion and marriage quality in both the control group and the patient group, but support outside the family was positively associated with marriage quality only in the patient group.

**Table 3:** **Correlation between Social Support and Family Functioning, Marriage Quality and Negative Emotion**

		**FACES II-CV**				**ENRICH**		**SAS-CV**		**SDS-CV**	
**Measure**	**Subscale**	**Family cohesion**		**Family adaptation**		**Marital quality**		**Anxiety**		**Depression**	
	**Group**	**Control Patients**		**Control Patients**		**Control Patients**		**Control Patients**		**Control Patients**	
**PSSS**	Support within family	0.48**	0.26*	0.11	0.04	0.37**	0.59**	−0.16	0.07	−0.26*	−0.18
	Support outside family	0.16	0.15	−0.19	0.05	0.11	0.46**	−0.07	0.07	−0.26**	−0.11
**SSRS**	Emotional support	0.16	0.31**	0.25*	−0.15	−0.01	0.08	−0.16	0.08	0.03	0.09
	Instrumental support	0.07	0.13	0.26*	0.18	−0.02	0.13	−0.08	−0.30*	0.28**	0.22

(**P < 0.001), (*P < 0.005).

Both emotional and instrumental support were positively associated with family adaptation in the control group, while they were not related with family adaptation in patients. Emotional support was positively related with family cohesion in patients.

Support within and outside the family were negatively associated with depression only in the control group. Instrumental support was negatively associated with anxiety in patients but positively associated with depression in healthy controls (Table 3).

### Multiple linear regression analysis

In the control group, support within the family significantly predicted family cohesion (R^2^ = 0.365). Support within and outside family, instrumental support and depression significantly predicted family adaptation (R^2^ = 0.371). Support within the family significantly predicted marital quality (R^2^ = 0.173). In the patient sample, support within the family and emotional support significantly predicted family cohesion (R^2^ = 0.240), instrumental support and depression significantly predicted family adaptation (R^2^ = 0.229). Finally, support within the family and emotional support predicted marriage quality (R^2^ = 0.511) (Table 4).

**Table 4:** **Results of multiple linear regression analysis**

**Dependent variables**	**Independent variables**	**Unstandardized Coefficients**		***SE***		**Standardized Coefficients**		***t***		***Sig.***		***F***		***Sig.***		***R*** ^**2**^	
	**Group**	**Control**	**Patient**	**Control**	**Patient**	**Control**	**Patient**	**Control**	**Patient**	**Control**	**Patient**	**Control**	**Patient**	**Control**	**Patient**	**Control**	**Patient**
Family cohesion	Constant	63.256	26.341	10.890	9.236			5.809	2.852	<0.001	0.006	5.455	3.005	<0.001	0.013	0.365	0.240
	Support within family	1.124	0.893	0.254	0.332	0.548	0.437	4.417	2.688	<0.001	0.009						
	Support outside family	0.057	−0.137	0.140	0.199	−0.149	−0.116	−0.408	−0.690	0.684	0.493						
	Emotional support	0.232	0.809	0.171	0.266	0.159	0.337	1.356	3.042	0.180	0.004						
	Instrument support	0.247	0.203	0.345	0.598	0.081	0.042	0.715	0.339	0.478	0.736						
	Anxiety	−0.111	−0.128	0.187	0.206	−0.073	−0.120	−0.595	−0.620	0.554	0.538						
	Depression	−0.242	0.201	0.138	0.175	−0.218	0.213	−1.760	1.143	0.084	0.258						
Family adaptation	Constant	51.370	64.717	11.155	10.151			4.605	6.375	<0.001	<0.001	5.597	2.820	<0.001	0.018	0.371	0.229
	Support within family	0.731	−0.146	0.261	0.365	0.346	−0.066	2.806	−0.400	0.007	0.691						
	Support outside family	−0.353	−0.047	0.143	0.219	−0.296	−0.037	−2.461	−0.216	0.017	0.830						
	Emotional support	0.070	−0.279	0.175	0.292	0.046	−0.119	0.398	−0.953	0.692	0.345						
	Instrumental support	0.878	1.327	0.354	0.657	0.280	0.251	2.481	2.018	0.016	0.048						
	Anxiety	0.149	0.059	0.192	0.227	0.095	0.051	0.779	0.261	0.439	0.795						
	Depression	−0.531	−0.471	0.141	0.193	−0.464	−0.460	−3.765	−2.445	<0.001	0.018						
Marital quality	Constant	333.234	253.743	49.382	31.111			6.748	8.156	<0.001	<0.001	1.989	9.909	0.082	<0.001	0.173	0.511
	Support within family	3.068	4.674	1.153	1.120	0.376	0.545	2.659	4.174	0.010	<0.001						
	Support outside family	−0.331	0.334	0.635	0.670	−0.072	0.067	−0.522	0.498	0.604	0.620						
	Emotional support	0.263	2.769	0.776	0.896	0.045	0.308	0.339	3.090	0.736	0.003						
	Instrumental support	0.046	0.584	1.566	2.014	0.004	0.029	0.030	0.290	0.936	0.773						
	Anxiety	−1.165	−0.639	0.848	0.695	−0.192	−0.143	−1.373	−0.920	0.175	0.361						
	Depression	0.280	−0.804	0.624	0.591	0.063	−0.204	0.449	−1.361	0.655	0.179						

## Discussion

Consistent with the first hypothesis, PWE reported higher levels of anxiety and depression, and had lower family cohesion and marriage quality compared with healthy controls. Previous studies have found that compared with the general population, PWE are much more likely to report symptoms of depression [22,25,26,36] or anxiety [37]. Anxiety and depression have been associated with poor quality of life of PWE [7]. The present study therefore supports previous evidence that PWE have higher levels of anxiety and depression than healthy controls, suggesting clinical and psychological interventions should target the psychological wellbeing of PWE.

The findings of the present study support the second hypothesis that PWE received less emotional and instrumental support than healthy controls, coming both from within and outside the family. Previous research examining social support in those with chronic diseases has yielded mixed results. Some studies have indicated that those with chronic illness received more social support [38,39] and that people under stressful conditions are more likely to search for help and therefore receive more social support [12,40]. Other studies however have found that those with chronic disease receive less social support [10,13]. A previous study showed that hemodialysis patients and their spouses had higher levels of stress reaction but did not receive more social support coming from family and outside family [13].

There could be several possible reasons for this inconsistency. Illness could have a negative impact on the patients’ social life and they may have reduced their social activities or isolated themselves from others [41]. It is also possible that perceptions of stigma mean that patients feel less able to ask for help and support. European surveys indicate that more than 50% of PWE feel stigma [42]. Studies in China reported stigma to be felt by 71% PWE in urban areas and by 89% PWE in rural areas [43]. As stigma is the major factor limiting social activities of PWE and lowing their quality of life [44,45], further studies should pay more attention to the relationship between stigma, social support and family life of PWE.

The present study found that only instrumental (financial or material) support predicted anxiety in patients, and not other kinds of social support. Depression in patients was not predicted by perceived social support. Previous research has found that lower socioeconomic status and unemployment is negatively associated with QOL in PWE [22], which supports the finding that instrumental support could be important in predicting psychological wellbeing in this population. Recently, scholars have argued that different sources of support may induce different consequences, and pointed out that received support may not be beneficial because it is associated with a threat to self-esteem or one’s sense of independency, which in turn offsets any benefits of received support [14,40,46]. These findings reveal a complex picture of the health implications of social support.

Both marriage and family are major sources of social support and predictors of health of patients with chronic diseases [23]. However, few studies about family functioning and marital quality of PWE had been conducted [20,24,47]. The results of the present study showed that patients had lower levels of family cohesion, family adaptation and marital quality compared with healthy controls. These results support the idea that marriages that involve PWE are more likely to experience difficulties, with patients with both epileptic seizures and nonepileptic seizures reporting they had family dysfunction [23,24]. Seizures and hospitalization also had a negative impact on the family life of PWE and family members [25,47]. In addition, the current study examined family adaptation of PWE and found depression negatively affected family adaptation in PWE. Family adaptation means quality and expression of the family’s leadership, organization, roles and relationship rules. Good adaptation of families means adapting in predictable ways in response to life changes and stress events [20,48]. Lower family adaptation and higher depression may explain the reason that PWE are more likely to have marriage difficulties.

Few studies have focused on the relationship between family functioning, marital quality and social support of PWE. The current results indicate that support within the family was positively related with family cohesion and marriage quality in both PWE and healthy control, while support outside the family was positively related with marriage quality only in PWE. Emotional support was positively related with family cohesion in the patient group. Those patients with PWE who were married with poor social support were less likely to report excellent self-rated health status and better life satisfaction [45]. It should be noted that emotional and instrumental support were positively related with family adaptation in healthy people, but they were not related with family adaptation in PWE. A previous study found that both emotional and instrumental supports were not related with family functioning and marital quality in patients during dialysis [13]. It appears that it is necessary to clarify what kinds of social supports are useful to PWE and other patients with chronic diseases.

This study has several limitations, including the relatively small sample size, self-report method and the inclusion criteria of well controlled seizure subjects which limits the generalizability of the findings. Future studies should test the conclusions in a larger sample size. Further studies should pay attention to demographic variables such as gender, age and education and psychological variable such as stigma, self-conception and the ways in which these affect family and marital quality.

## Conclusions

The current study explored anxiety, depression, social support and family life of PWE. Findings suggest that PWE in China had higher levels of anxiety and depression, less satisfaction with family functioning and marital life, and less social support than healthy controls. Social support did not predict depression in patients however, and only instrumental support predicted anxiety in patients. The present study explored the predictive value of social supports and negative emotion on family functioning and marital quality in patients and healthy controls. The findings support the conclusion that emotional support coming from family members and depression predicts family functioning and marital quality of PWE. These findings suggest that enhancing family and emotional supports and decreasing depression could promote the family cohesion and marital quality of PWE, and instrumental support may play a role in decreasing anxiety.
